# Phosphorylation Modulates the Subcellular Localization of SOX11

**DOI:** 10.3389/fnmol.2018.00211

**Published:** 2018-06-19

**Authors:** Elli-Anna Balta, Marie-Theres Wittmann, Matthias Jung, Elisabeth Sock, Benjamin Martin Haeberle, Birgit Heim, Felix von Zweydorf, Jana Heppt, Julia von Wittgenstein, Christian Johannes Gloeckner, Dieter Chichung Lie

**Affiliations:** ^1^Institute of Biochemistry, Friedrich-Alexander-Universität Erlangen-Nürnberg, Erlangen, Germany; ^2^Center for Ophthalmology, Institute for Ophthalmic Research, University of Tübingen, Tübingen, Germany; ^3^DZNE-German Center for Neurodegenerative Diseases, Tübingen, Germany

**Keywords:** SOX11, transcription factor phosphorylation, subcellular localization, neurogenesis, cancer, intellectual disability

## Abstract

SOX11 is a key Transcription Factor (TF) in the regulation of embryonic and adult neurogenesis, whose mutation has recently been linked to an intellectual disability syndrome in humans. SOX11’s transient activity during neurogenesis is critical to ensure the precise execution of the neurogenic program. Here, we report that SOX11 displays differential subcellular localizations during the course of neurogenesis. Western-Blot analysis of embryonic mouse brain lysates indicated that SOX11 is post-translationally modified by phosphorylation. Using Mass Spectrometry, we found 10 serine residues in the SOX11 protein that are putatively phosphorylated. Systematic analysis of phospho-mutant SOX11 resulted in the identification of the S30 residue, whose phosphorylation promotes nuclear over cytoplasmic localization of SOX11. Collectively, these findings uncover phosphorylation as a novel layer of regulation of the intellectual disability gene Sox11.

## Introduction

SOX11 (SRY-related HMG-box 11) is a member of the SoxC Transcription Factor (TF) family. The SoxC family, which in addition to SOX11 comprises SOX4 and SOX12, potently regulates the development of the mammalian nervous system (Wegner, 2011; Kavyanifar et al., 2018). Sox11 expression is strongly associated with neurogenic activity and is with regard to the central nervous system (CNS) largely confined to the embryonic (Uwanogho et al., 1995; Hargrave et al., 1997; Rimini et al., 1999; Sock et al., 2004) and adult germinal zones (Mu et al., 2012; Wang et al., 2013).

Mutations in SOX11 were found to be causal for Coffin-Siris Syndrome, a congenital disorder associated with intellectual disability and microcephaly, which illustrates the importance of SOX11 for human CNS development and underlines the need to understand SOX11’s regulation (Tsurusaki et al., 2014; Hempel et al., 2016). SOX11 controls multiple processes in neurogenesis including precursor survival, proliferation, fate commitment, axonal growth, dendritic morphogenesis, migration and maturation (Bergsland et al., 2006; Hide et al., 2009; Lin et al., 2011; Mu et al., 2012; Wang et al., 2013; Hoshiba et al., 2016). Recent findings indicate that not only the expression of SOX11 itself but also its transient activity is critical to ensure the precise execution of the neurogenic program (Hoshiba et al., 2016). Thus, Sox11 ablation in developing cortical neurons results in premature development of an elaborate dendrite compartment and migration deficits; whereas, Sox11 overexpression does not affect neuronal migration but severely impairs dendritic development (Hoshiba et al., 2016).

SOX11 plays not only a major role in neurodevelopment, but may also be important for regeneration in the peripheral and CNS (Jankowski et al., 2009; Kuwajima and Mason, 2017; Norsworthy et al., 2017; Struebing et al., 2017). Thus, SOX11 knockdown impairs peripheral nerve regeneration (Jankowski et al., 2009), while SOX11 overexpression triggers axonal growth and enhances the survival of a distinct subset of retinal ganglion cells following injury (Norsworthy et al., 2017).

The regulation of SOX11 has been primarily explored on the level of gene expression. The chromodomain helicase domain protein Chd7 was described to regulate Sox11 expression in adult neural stem cells via remodeling of the Sox11 promoter to an open chromatin state (Feng et al., 2013). It has also been reported that a complex comprising the chromatin remodeling factor BAF and the neurogenic TF PAX6 activates Sox11 expression in adult neural stem cells (Ninkovic et al., 2013). There is growing evidence that the stability, activity and cellular distribution of Sox proteins are potently regulated by post-translational modifications (Rehberg et al., 2002; Hattori et al., 2006; Baltus et al., 2009; Swartling et al., 2009; Lai et al., 2011; Fang et al., 2014). Such regulation by post-translational modifications is best documented for the SoxB family TF SOX2 (Baltus et al., 2009; Fang et al., 2014). It has, for example, been shown that the balance between methylation and phosphorylation of SOX2 dictates the choice between differentiation and maintenance of embryonic stem (ES) cells (Fang et al., 2014). Methylation results in degradation of SOX2, thereby promoting differentiation of ES cells, while phosphorylation stabilizes SOX2 and promotes pluripotency. Acetylation-mediated nuclear export of SOX2 was also shown to tilt the balance between pluripotency and differentiation towards the latter (Baltus et al., 2009). There is some evidence that SOX11 may be modified and regulated by post-translational modifications. SOX11 was found to be located in different cellular compartments in pathological contexts. SOX11’s subcellular localization is considered a prognostic marker in mantle cell lymphoma, in which nuclear localization of SOX11 suggests good prognosis while cytoplasmic localization is associated with shorter survival (Wang et al., 2008). A recent study reported that SOX11’s subcellular localization in retinal ganglion cells is modulated by SUMOylation (Chang et al., 2017). Another study reported that SOX11 biochemically interacts with the Nemo-like kinase in developing *Xenopus* (Hyodo-Miura et al., 2002), raising the possibility that SOX11 is also modified by phosphorylation. Here, we investigated whether SOX11 subcellular localization changes during the course of mammalian neurogenesis and aimed to reveal posttranslational modifications (PTMs) that influence SOX11’s subcellular localization.

## Materials and Methods

### Animal Experiments

All animal experiments performed in accordance with the European Communities Council Directive (86/609/EEC) and approved by the governments of Upper Bavaria and Middle-Franconia. C57Bl/6NRj mice were obtained from Janvier Labs (Le Genest-Saint-Isle, France). The Sox11^LacZ/wt^ mice were previously described (Sock et al., 2004). E18.5 Sox11 knock out mice (Sox11^LacZ/LacZ^) were obtained from breedings of Sox11^LacZ/wt^ mice. All mice were group-housed under a 12 h light/dark cycle with *ad libitum* access to food and water.

### Tissue Preparation, Immunofluorescence Staining and Imaging

For histological analyses of adult mice, 2-month old male and female animals were killed with CO_2_ and transcardially perfused with phosphate-buffered saline (PBS, pH = 7.4) followed by 4% paraformaldehyde (PFA) in Phosphate buffer (pH = 7.4). Brain tissue was post-fixed in 4% PFA overnight and dehydrated in 30% sucrose solution before slicing at a sliding microtome (Leica Microsystems, Wetzlar, Germany). Immunofluorescence stainings were carried out on free-floating sections. Slices were washed five times in Tris-buffered saline (TBS; 25 mM Tris/HCl, 137 mM NaCl, 2.6 mM KCl). Slices were incubated in blocking solution (TBS, 10% normal donkey serum, 0.25% TritonX-100) for 1 h and subsequently incubated in blocking solution containing primary antibodies (Table 1) for 72 h. Slices were washed six times in TBS and incubated with fluorophore-coupled secondary antibodies (Table 2) overnight. The following day, brain slices were subjected to nuclear staining with DAPI (2 μM in TBS), washed three times and mounted in Aqua Polymount (Polysciences, Warrington, PA, USA).

**Table 1:** Primary antibodies.

Antigen	Species	Company	Working dilution	Catalog number	RRID
SOX11	Rabbit	Abcam	1:500	ab134107	AB_2721126
Doublecortin (DCX)	Goat	Santa Cruz	1:500	SC8066	AB_2088494
GFP	Chicken	Aves Labs	1:500	GFP-1020	AB_10000240
p-CREB(S133)	Rabbit	Cell Signaling	1:500	9198S	AB_2561044
GAPDH	Mouse	Santa Cruz	1:500	SC32233	AB_627679
αTUBULIN	Mouse	Sigma	1:1000	T9026	AB_477593
pRNA polymerase II	Rabbit	Abcam	1:500	ab5095	AB_304749

**Table 2:** Secondary antibodies.

Antibody	Host	Company	Working dilution	Catalog number	RRID
Alexa488-coupled anti goat IgG	Donkey	Invitrogen	1:1000	A11055	AB_2534102
Cy3-coupled anti rabbit IgG	Donkey	Jackson	1:1000	711-165-152	AB_2307443
Cy5-coupled anti mouse IgG	Donkey	Jackson	1:1000	715-175-151	AB_2340820
Alexa488-coupled anti chicken IgG	Donkey	Jackson	1:1000	20166	AB_10854387
Horseradish peroxidase (HRP)-coupled anti mouse IgG	Goat	Jackson	1:1000	115-035-003	AB_10015289
Horseradish peroxidase (HRP)-coupled anti rabbit IgG	Goat	Jackson	1:1000	111-035-003	AB_2313567

For histological analyses of the embryonic mouse brain, timed pregnant mice were killed by cervical dislocation and embryos were removed. Embryonic day (E) 11.5 and E13.5 embryos underwent overnight fixation in 4% PFA. For the E15.5 time point, heads were fixed overnight in 4% PFA. For the E18.5 and postnatal day 0 (P0) time points, brains were dissected and fixed overnight in 4% PFA. The tissue was transferred to 30% sucrose in PBS overnight for dehydration. Embryonic tissues were embedded in freezing media (Jung, Nussloch). Embryonic tissue was cut in 10 μm thin sections with a cryotom (Leica Microsystems, Wetzlar). Sections were transferred to slides and dried for 2 h at room temperature and stored at −80°C. Sections were washed with PBS, treated with 50 mM citrate buffer at 70°C for 3 min for antigen retrieval. Tissue was permeabilized in 0.1% Triton-X/PBS and blocked with blocking solution (10% FCS, 1% BSA in PBS) at room temperature for 2 h in a wet chamber. Sections were incubated overnight with primary antibodies diluted in blocking solution at 4°C. Slides were washed with PBS, incubated with secondary antibodies diluted in blocking solution for 2 h at room temperature and washed once with PBS. Nuclei were stained with DAPI for 2 min. After additional washing with PBS 2× for 10 min, slides were mounted with 50 μl Mowiol (Sigma-Aldrich) and stored at 4°C.

For imaging of both adult and embryonic slices, confocal single plane and z-projection images were generated at a Zeiss LSM 780 equipped with four lasers (405, 488, 559 and 633 nm) and 40× and 63× objectives. Image processing was done in Fiji ImageJ (Schindelin et al., 2012).

Nuclear vs. cytoplasmic localization was counted on the basis of SOX11 and DAPI stainings. Assessment was verified by analysis via line intensity plots. We analyzed three samples (*n* = 3) per developmental time-point. In each sample at least 100 cells were evaluated.

### Tissue Culture, Transfection, Immunofluorescence Stainings and Imaging

For transfection, HEK 293T cells (ATCC, Wesel, Germany; CRL-3216) were seeded in 24-well plates at a density of 60,000 cells per well. Twenty-four hours after, the cells were transfected with jetPEI (Polyplus transfection, 101–10N) according to the manufacturer’s protocol (0.5 μg DNA/0.5 μl PEI reagent/well). Forty-eight hours after transfection cells were washed once with PBS, fixed with 4% PFA for 10 min, washed two times with PBS and incubated for 1 h in blocking solution. Cells were incubated overnight with primary antibodies diluted in blocking solution. Cells were washed three times in PBS and incubated with fluorophore-coupled secondary antibodies for 2 h, stained with DAPI for 10 min, washed three times and finally mounted in Aqua Polymount (Polysciences, Warrington, PA, USA) for imaging. Single plane and z-projection images were taken on a Zeiss LSM 780 confocal microscope equipped with four lasers (405, 488, 559 and 633 nm) and 40× and 63× objectives. Image processing was done in Fiji ImageJ (Schindelin et al., 2012). Nuclear vs. cytoplasmic localization was counted on the basis of SOX11 and DAPI stainings. Assessment was verified by analysis via line intensity plots. We analyzed three samples (*n* = 3) per condition. In each sample at least 50 cells were evaluated.

### Line Intensity Plots

Comparative line intensity plots of the fluorescence of the nuclear label DAPI and of the immunofluorescence of SOX11 were used to validate SOX11’s subcellular localization. The data plotted on the line intensity plots were produced by using the Plot Profile function of Image J (Schindelin et al., 2012) on a single plane z-stack of confocal microscopy pictures (Supplementary Figures S1F–K).

### Reporter Assay (Luciferase Assay)

For the reporter assays, HEK 293T cells were seeded and transfected as described above with equal amounts (0.05 μg/well) of the expression vectors (C3, C3-Sox11p^WT^, C3-Sox11p^N1W9^ and C3-Sox11p^M1W9^) together with the Sox11-responsive minimal promoter-Luciferase reporter construct (0.05 μg/well) and a Renilla–construct under the control of the human elongation factor one promoter (0.005 μg/well; Lie et al., 2005). Forty-eight hours after transfection the cells were analyzed via the Promega dual luciferase kit and a Centro LB 960 luminometer. Luciferase assay was performed from three biological replicates. Significance was tested using Student’s unpaired *t*-test (*p < 0.05, **p < 0.01, ***p < 0.001).

### Plasmids

Mouse wildtype (WT) and mutant Sox11 were cloned into the pEGFP-C3 plasmid (Clontech) to generate N-terminal fusion proteins with GFP to ensure detection of the mutants in case the antibody failed to recognize them. The Sox11 mutants (C3-Sox11p^MIMIC^ and C3-Sox11p^NON^) were synthesized by Thermo Fisher Scientific GENEART GmbH and cloned into the pEGFP-C3 plasmid. All other mutants were obtained by restriction enzyme digestions and combinatorial ligation of the C3-Sox11p^MIMIC^, C3-Sox11p^NON^ mutants and WT Sox11. Non GFP fused Sox11 (expressed of the pCAG–Sox11–IRES–GFP plasmid; Mu et al., 2012) and SOX11-GFP fusion protein showed similar nuclear and cytoplasmic distribution in transiently transfected HEK293T cells, indicating that the N-terminal GFP fusion did not alter subcellular distribution of SOX11 localization (Supplementary Figures S2D–F). For phosphosite mapping, Sox11 was cloned into the pDEST-NSF vector described in Gloeckner et al. (2007) producing an N-terminally Strep-Flag tagged Sox11. The minimal SOX11 responsive promoter-Luciferase comprises a TATA box and multiple repeats of Sox11 binding motifs upstream of the luciferase gene (Kuhlbrodt et al., 1998).

### Nuclear—Cytoplasmic Fractionation and Western Blot

Timed pregnant mice were killed by cervical dislocation and the brains from E15.5 and E18.5 embryos were immediately processed. Brains were disrupted and lysed in Buffer A (10 mM Hepes, 1 mM EDTA, 0.1 mM EGTA, 10 mM KCl, 1 mM PMSF, 1 mM DTT, 1 μg/μl protease inhibitor cocktail-EDTAfree-Roche) with the use of a tissue homogenizer. 0.1% NP-40 was added to the homogenates, which were then vortexed shortly and incubated on ice for 10 min. To separate cytoplasmic from the nuclear fraction, samples were centrifuged for 5 min at 4°C at 10,000 *g*. The supernatant, which corresponds to the cytoplasmic fraction, was equally distributed between: (i) a tube that contained lambda phosphatase (λPP) for removal of phosphorylations (lambda phosphatase, P0753S, New England Biolabs, applied according to the manufacturer’s instructions) to generate λPP treated cytoplasmic extract (C_λPP_); and (ii) a tube that contained 1 μg/μl Phospho Stop (Phosphatase inhibitor cocktail, Sigma Aldrich) for preservation of phophorylations to generate PS treated cytoplasmic extract (C_PS_). The pellet was washed with Buffer A followed by centrifugation for 5 min at 4°C at 10,000 *g* and removal of the supernatant. To generate nuclear extracts, the pellet was disrupted with the tissue homogenizer in RIPA buffer (50 mM Tris pH 8, 150 mM NaCl, 1 mM EDTA, 1% NP-40, 0.1% SDS, 0.5% S-DOC, 1× protease inhibitor cocktail-EDTAfree-Roche). Following 15 min incubation on ice, the samples were centrifuged for 5 min at 4°C at 14,000 *g*. The supernatant was equally distributed between: (i) a tube that contained λPP to generate λPP treated nuclear extract (N_λPP_); and (ii) a tube that contained 1 μg/μl Phospho Stop to generate PS treated nuclear extract (N_PS_; Supplementary Figure S3A).

For the fractionation of nuclear and cytoplasmic extracts from Neuro2A cells, 10^6^ cells were seeded per 10 cm dish. The next day, cells were transfected with C3-Sox11p^WT^, C3-Sox11p^N1W9^, C3-Sox11p^M1W9^, C3-Sox11p^W1N2W7^, or C3-Sox11p^W1M2W7^ using the jetPEI transfection reagent. Forty-eight hours after transfection, cells were washed with 1× PBS and collected using a cell scraper in 0.5 ml PBS. Cells were pelleted by centrifugation at 500 *g* for 5 min. The supernatant was discarded and cells were resuspended in 3× their volume of Buffer A. 0.1% NP-40 was added followed by vortex and 2 min incubation on ice. The samples were centrifuged (10,000 *g* for 5 min 4°C) and the supernatant (C = Cytoplasmic extracts) was transferred to a new tube. The pellets, containing the nuclei, were subsequently resuspended in 2× their volume of RIPA buffer and incubated on ice for 10 min followed by centrifugation at 14,000 *g* for 5 min at 4°C. The supernatants (N = Nuclear extracts) were then transferred to a new tube. Extracts were separated in a 10% SDS-PAGE gel. Gels underwent wet transfer onto a Nitrocellulose membrane. Membranes were blocked in 5% ^w/v^ skim milk (Sigma Aldrich) in TBS with 0.1% Tween 20 (TBS-T). Incubation with primary antibodies diluted in blocking solution was performed overnight at 4°C and was followed by washing with TBS-T. The appropriate secondary antibodies were diluted in blocking solutions and incubated with the membranes for at least 1 h at room temperature followed by washing with TBS-T. The membranes were visualized via Clarity Western Enhanced Chemiluminescence (ECL) Substrate (Bio-Rad) with ChemiDoc XRS+ System (Bio-Rad). Images were subsequently processed via ImageLab 5.2.1 Setup (Bio-Rad).

### Mass Spectrometry

For mass spectrometric phosophosite mapping, N-terminally Strep-Flag tagged Sox11 (NSF-tagged Sox11) was recombinantly expressed in either HEK293T or Neuro2a cells. Sox11 was immunoprecipitated via a FLAG tag using Flag M2 Sepharose (Sigma Aldrich, Taufkirchen, Germany) and eluted by 1× Laemmli buffer from the affinity resin. Eluates were separated on a 10% BisTris NuPAGE gel (Thermo Fisher Scientific, Dreieich, Germany). Parts of the gel corresponding to the expected molecular weight of Sox11 were excised cut into pieces and subjected to tryptic in gel proteolysis following standard protocols as described in Gloeckner et al. (2009). After extraction of the peptides from the gel plugs, phosphopeptides were enriched by TiO_2_ following protocols described in Gloeckner et al. (2010). After phosphopeptide enrichment, samples were analyzed either on an Orbitrap Fusion (Thermo Fisher Scientific, Dreieich, Germany) instrument or an Orbitrap Q Exactive Plus (Thermo Fisher Scientific, Dreieich, Germany) coupled to a RSLC3000 nano HPLC system (Thermo Fisher Scientific, Dreieich, Germany). All MS/MS samples were extracted and analyzed using Proteome Discoverer (Thermo Fisher Scientific, Bremen, Germany, version 1.4.1.14) with Mascot (Matrix Science, London, UK; version 2.5.1) as search engine. For samples derived from HEK293T, Mascot was set up to search the human subset of the swissprot database where the human Sox11 was replaced by the murine Sox11 sequence (version 2015-05, 20198 entries) with the proteolytic enzyme set to trypsin. Mascot was searched with a fragment ion mass tolerance of 0.60 Da and a parent ion tolerance of 10.0 PPM. Carbamidomethyl of cysteine was specified in Mascot as a fixed modification. Deamidation of asparagine and glutamine, oxidation of methionine and phosphorylation of serine, threonine and tyrosine were specified in Mascot as variable modifications. For samples derived from Neuro2a, the murine subset of SwissProt (version 2015-03), 16711 entries were used as database with the same settings as described above. For assessing the exact position of the phosphorylation sites within the peptide sequence, the Proteome Discoverer node for PhosphoRS (version 3.0) has been used (Taus et al., 2011).

### Conservation Analysis

BLAST COBALT (Altschul et al., 1990, 1997; Papadopoulos and Agarwala, 2007; Boratyn et al., 2013), NCBI (National Center for Biotechnology Information, U.S. National Library of Medicine8600 Rockville Pike, Bethesda MD, USA) was used to align sequences.

## Results

**Figure 1:**
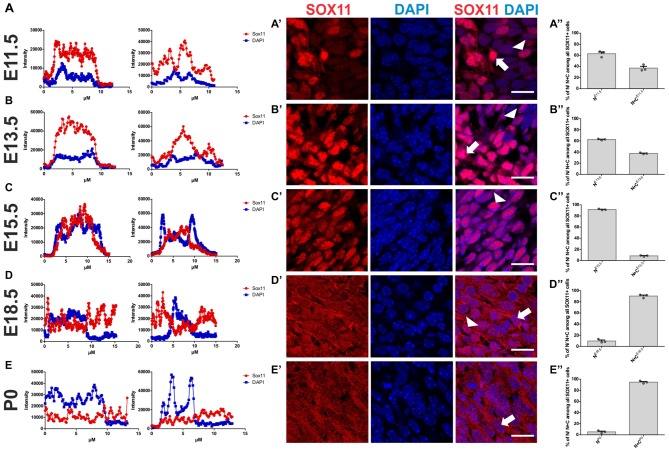
SOX11’s subcellular localization in embryonic neurogenesis. Representative line intensity plots of cells from E11.5 **(A)**, E13.5 **(B)**, E15.5 **(C)**, E18.5 **(D)** and P0 **(E)**. The blue line represents intensity of DAPI, the red line represents intensity of SOX11. Coronal sections of brains derived from E11.5 **(A′)**, E13.5 **(B′)**, E15.5 **(C′)**, E18.5 **(D′)** and P0 **(E′)** mice were stained for SOX11 (RED) and DAPI (blue). Arrows: cells with nuclear and cytoplasmic localization of SOX11; arrow heads: cells with exclusively nuclear localization of SOX11. Note the almost exclusive nuclear localization of SOX11 at E15.5. Scale bars: 20 μm. Graphs represent the percentage of cells with nuclear localization or nuclear and cytoplasmic localization of SOX11 over all the SOX11 positive cells counted from E11.5 **(A″)**, E13.5 **(B″)**, E15.5 **(C″)**, E18.5 **(D″)** and P0 **(E″)**. The data are presented as mean ± SEM.

**Figure 2:**
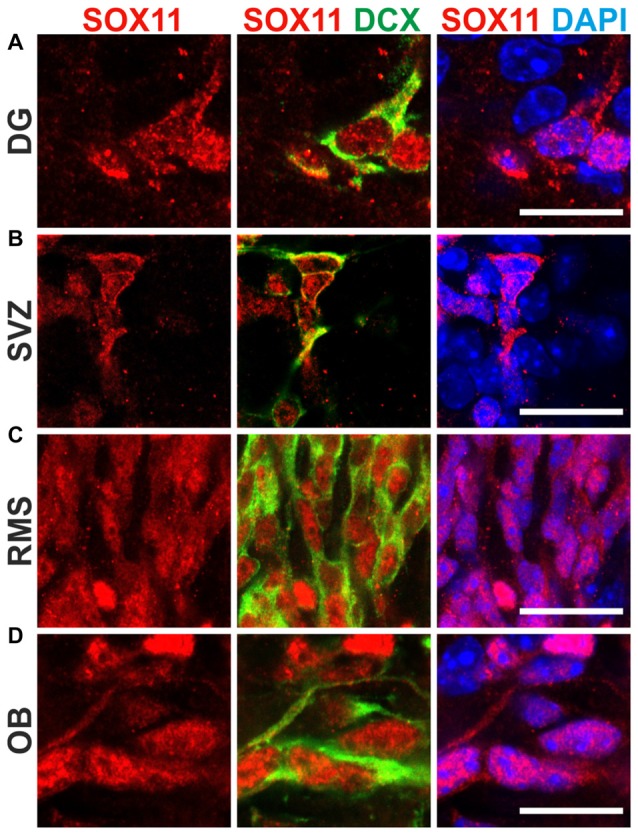
SOX11’s subcellular localization in adult neurogenesis. Immunofluorescent analysis of SOX11’s subcellular localization in the dentate gyrus (DG; **A)**, the subventricular zone (SVZ; **B)**, the rostral migratory stream (RMS; **C)** and the olfactory bulb (OB; **D)**. SOX11 (red), DCX (green), DAPI (blue). Scale bars: 20 μm.

To determine the subcellular localization of SOX11 during the course of neurogenesis, we conducted immunofluorescent stainings for SOX11 on the developing mouse cortex from E11.5 to P0 (Figure 1 and Supplementary Figure S1E). Specificity of the anti-SOX11 antibody had prior been validated by comparative immunofluorescent staining of E18.5 brain tissue from SOX11 WT and SOX11 knockout mice (Supplementary Figures S1A–D). On E11.5 and E13.5, we observed cells with an exclusively nuclear localization as well as cells with both nuclear and cytoplasmic localization of SOX11 (E11.5: nuclear 63.0%, nuclear and cytoplasmic 37.1%; E13.5: nuclear: 62.5%, nuclear and cytoplasmic: 37.5%; Figures 1A–B″). On E15.5, Sox11 localization was almost exclusively nuclear (nuclear: 91.5%, nuclear and cytoplasmic: 8.5%; Figures 1C–C″), whereas at later time points SOX11 localization was mainly nuclear and cytoplasmic (E18.5: nuclear 9.6%, nuclear and cytoplasmic 90.4%; P0: nuclear 5.4%, nuclear and cytoplasmic 94.6%; Figures D–D″,E–E″). The subcellular distribution of SOX11 differed between different areas of the developing brain. While Sox11 was almost exclusively nuclear in the developing cortex at E15.5 (Figures 1C–C″), developing subcortical regions frequently showed cells with a nuclear and cytoplasmic localization at the same developmental time-point (Supplementary Figures S2A–C’). We also examined SOX11’s subcellular localization in newly generated neurons in the two adult neurogenic niches (Figure 2). SOX11 is found in both nucleus and cytoplasm in cells of the subgranular zone of the dentate gyrus (DG), the subventricular zone (SVZ) of the lateral ventricles, the rostral migratory stream (RMS) and the olfactory bulb (OB; Figure 2). These results demonstrate that SOX11 can localize to the nucleus and to the cytoplasm during embryonic and adult neurogenesis.

**Figure 3:**
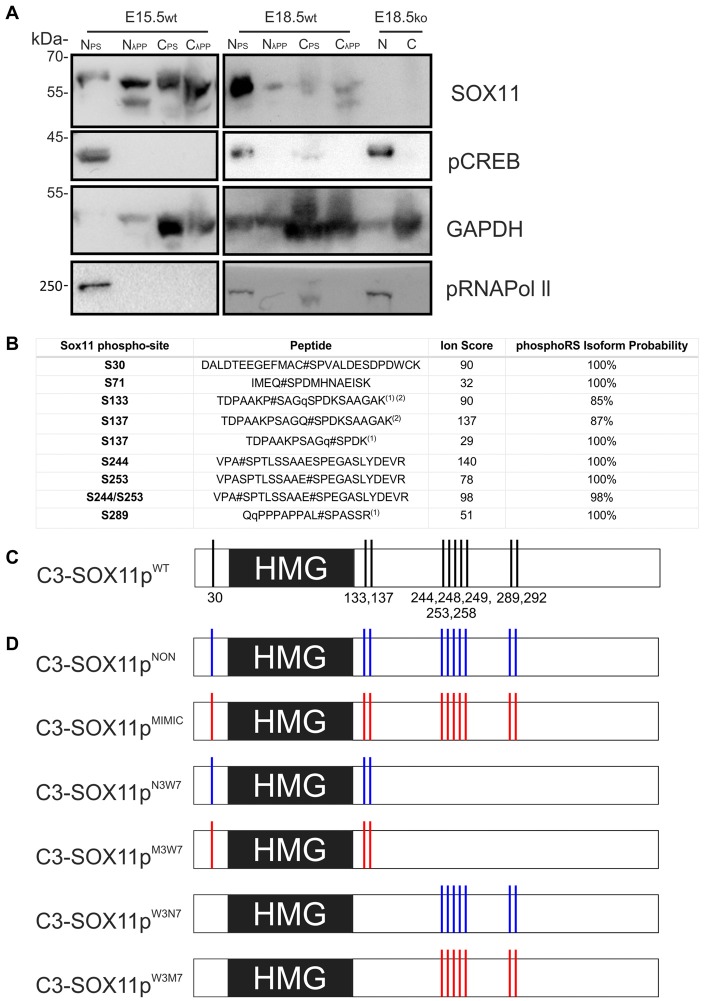
SOX11 is phosphorylated *in vivo* and has at least 10 phospho-serines *in vitro*. **(A)** Western Blot analysis of nuclear (N) and cytoplasmic (C) extracts treated with Phospho Stop (PS) or lambda phosphatase (λPP) from E15.5 and E18.5 WT mice (wt) and Sox11 knockout mice (ko). First row: blotting with an anti-SOX11 antibody. Second row: blotting with an anti-pCREB (S133) antibody verifies the functionality of λPP treatment and the enrichment of nuclear proteins. Third row: blotting with an anti-GAPDH validates the enrichment of cytoplasmic proteins. Fourth row: blotting with an anti-pRNA polymerase II validates enrichment of nuclear proteins and functionality of λPP. E18.5, knock out for Sox11, brain extracts (E18.5ko) validates that the bands in the WT brains are specific for SOX11. Note the different band pattern of the SOX11 signal between E15.5 nuclear extracts treated with PS and λPP. The SOX11 band pattern also appears to be changed by phosphatase treatment in E15.5 and E18.5 cytoplasmic extracts. **(B)** Identification of SOX11 phosphorylation sites by mass spectrometry. The table reports the SOX11 site, the peptide sequence, ion score and the phosphoRS metanalysis to identify the exact sites within the peptide sequence based on the MS/MS spectra. Peptides with highest site probabilities/ion scores have been selected from Supplementary Table S2 and contain peptides from both cell lines (N2A and HEK293T). All phosphopeptides were identified in both lines: (1) peptide with the highest peptide score contained a deubitquitination on Q(11)/Q(2) indicated by a small q; (2) peptides contain one missed cleavage. **(C)** Schematic representation of the putative phosphorylated serines on SOX11 protein. **(D)** Schematic representation of SOX11 mutants in which the serine residues that have been replaced by Alanine (NON-phosphorylatable amino acid) are marked with blue while the residues replaced by Aspartate (amino acid that MIMICS phosphorylation) are marked with red.

Phosphorylation is a powerful post-translational regulator of protein function, stability and localization (Rehberg et al., 2002; Hattori et al., 2006; Baltus et al., 2009; Swartling et al., 2009; Lai et al., 2011; Fang et al., 2014). To determine whether SOX11 is modified by phosphorylation *in vivo*, we analyzed nuclear and cytoplasmic protein extracts from E15.5 and E18.5 WT mouse brain following treatment with phosphatase inhibitors or λPP by Western Blot. Enrichment of nuclear and cytoplasmic proteins was validated by analysis of phosphorylated RNA Polymerase II and GAPDH, respectively. Analysis of protein extracts with a previously validated antibody against the S133 phosphorylated form of the TF CREB demonstrated that the phosphatase inhibitor and λPP treatments preserved or removed protein phosphorylation, respectively. Specificity of the band detected by the SOX11 antibody was validated by analysis of nuclear and cytoplasmic protein extracts from E18.5 Sox11 knockout mouse brains. SOX11 showed a different migration behavior between phosphatase inhibitor treated extracts and λPP treated extracts, which was most apparent in nuclear extracts from the E15.5 brain. Here, the SOX11-specific antibody detected a single band at 60 kDa in phosphatase inhibitor-treated extracts, but two defined lower molecular weight bands in phosphatase-treated extracts (Figure 3A). This clear differential phosphatase-dependent migration behavior demonstrates that SOX11 can *in vivo* be modified by phosphorylation. Phosphatase-treatment of cytoplasmic extracts from E15.5 and E18.5 appeared to generate a similar SOX11 migration pattern of two lower molecular weight bands (Figure 3A and Supplementary Figures S3B–G) suggesting that not only nuclear but also cytoplasmic SOX11 is modified by phosphorylation. Whether nuclear and cytoplasmic SOX11 harbor similar or distinct phosphorylations, however, could not be determined in this assay.

To identify phosphorylated residues, mass spectrometry (MS) analysis was performed from extracts from HEK293T and Neuro2a cells overexpressing N-terminally Strep-Flag tagged Sox11. This analysis revealed five different phosphopeptides indicating that *in vitro*, SOX11 protein can be phosphorylated at multiple residues (Figure 3B). The first two phosphopeptides flank the DNA-binding HMG-box, with the first phosphorylated serine (S30) being located proximal and the next two phosphorylated serines (S133, S137) being located immediately distal to the DNA-binding HMG-box. S137 was mapped by PhosphoRS (Taus et al., 2011) with significant scores. It is, however, of note that the exact sites could not be mapped with absolute confidence since S133 is in close proximity and the phosphorylated residue was not covered by neither the y-ion series nor the b-ion series of the MS2 spectra (Supplementary Table S1). We found at least three phosphorylations within the Sox11 C-terminus covered by two phosphopeptides. Though the sequence was not fully covered by the MS2 spectra, PhosphoRS scoring suggested phosphorylation at the residues S244 and S253. An additional site, either on S289, S292 or S293, was identified in close proximity to the transactivation domain. Here, PhosphoRS scoring indicates phosphorylation at S289. Figure 3C summarizes the localization of the phosphorylated residues in relation to the HMG-box.

**Figure 4:**
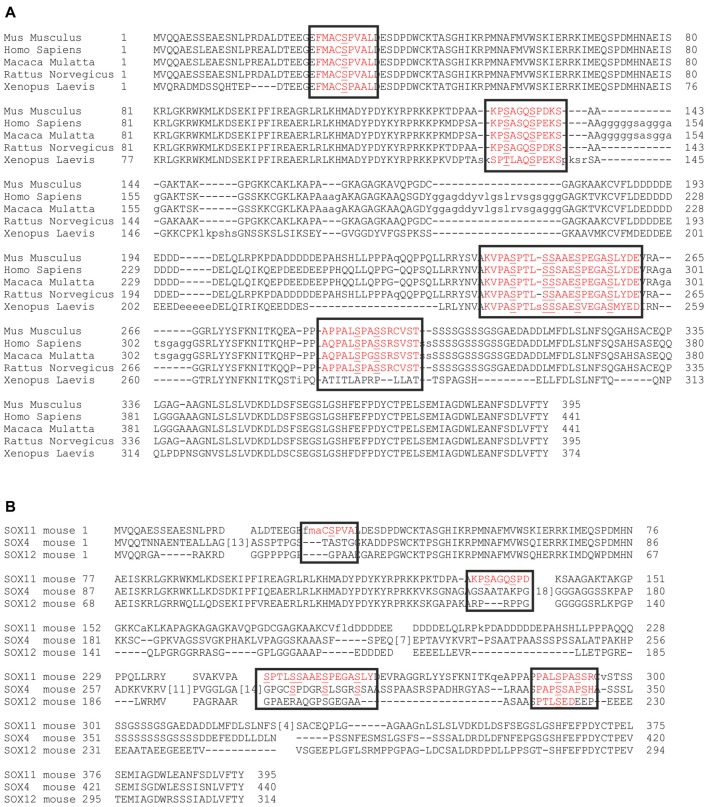
Conservation of the phosphorylatable serines. Alignment of SOX11 amino acid sequence from different species shows the conservation of the phosphorylatable serines **(A)**. Alignment of murine amino acid sequences of SOX11 (row1), SOX4 (row2) and SOX12 (row3) shows the conservation of the phosphorylatable serines among the SoxC family **(B)**. Black boxes indicate the regions of interest. Red letters indicate the occurrence of conservation. Note that the phosphorylatable serines surrounding the HMG-box (S30, S133, S137) are unique to SOX11.

Comparison of SOX11 between different species showed that the phosphorylated serine residues were conserved in mammals and most of them also in *Xenopus*, suggesting that their phosphorylation status may be involved in the regulation of SOX11 (Figure 4A). We further analyzed whether the phosphorylated serine residues are conserved between members of the SoxC family (Figure 4B). The two phosphorylated serine residues S289 and S292 together with their surrounding sequences are present in SOX4, while only S289 is found in SOX12. The sequence around the serine residues S244 and S253 is absent from SOX12 and only partially present in SOX4. Interestingly, the putatively phosphorylated serine residues flanking the HMG-box (S30, S133, S137) as well as their surrounding sequences are absent from both SOX4 and SOX12, suggesting that phosphorylation at these residues might constitute a regulatory mechanism unique to SOX11.

**Figure 5:**
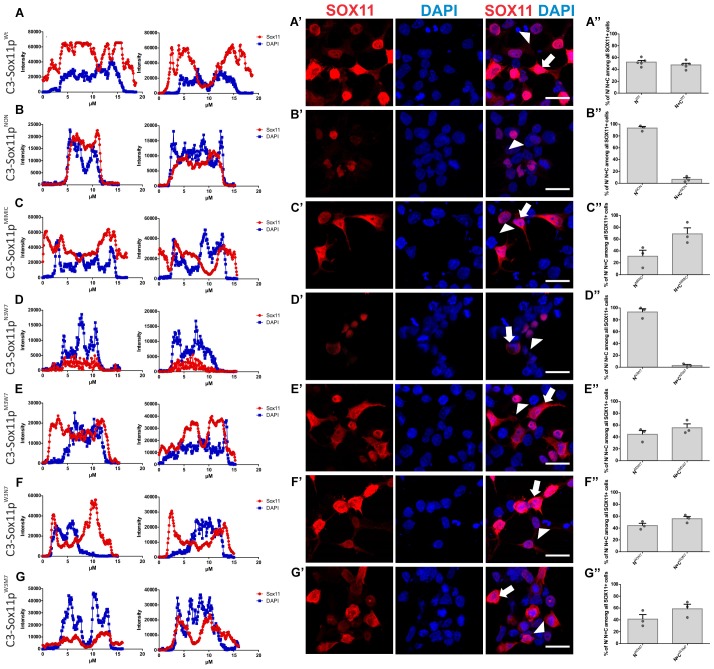
SOX11’s non-phosphorylatable form leads to nuclear localization. **(A–G)** Representative line intensity plots of HEK293T cells transfected with SOX11 wildtype (WT) and mutants. The blue line represents DAPI intensity, the red line represents SOX11 intensity. **(A′–G′)** Immunostaining for SOX11 (red) and DAPI (blue) of cells transfected with SOX11 WT and mutants were to determine the subcellular localization of SOX11 phospho-mutants. Note that the C3-Sox11p^NON^ **(B′)** and C3-Sox11p^N3W7^ **(D′)** are almost exclusively localized in the nucleus. Arrow: cell with nuclear and cytoplasmic localization of SOX11. Arrow head: cell with only nuclear localization of SOX11. Scale bars: 20 μm. **(A″–G″)** Percentage of cells with nuclear localization (N) or nuclear and cytoplasmic (N + C) localization of the different SOX11 mutants.

Next, we investigated the impact of phosphorylation on SOX11’s subcellular localization. We first mutated all phosphorylatable serines either to an amino acid (Alanine) that cannot be phosphorylated (C3-Sox11p^NON^) or to an amino acid (Aspartate) that mimics phosphorylation (C3-Sox11p^MIMIC^; Figure 3D). Given the bias for mono phosphorylated peptides in the TiO2 enriched fractions and the likely appearance of different phospho-isoforms of one peptide, we considered also serines with lower phosphorylation probabilities in the mutational approach in order not to miss functional relevant sites. The impact of these mutations on the localization of SOX11 was tested by transfection of the mutants into HEK293T cells. WT Sox11 (C3-Sox11p^WT^) and Sox11p^MIMIC^ were localized in both nucleus and cytoplasm while the Sox11p^NON^ was almost exclusively localized in the nucleus as indicated by the immunofluorescent stainings, the intensity plots and the counting (WT: Nuclear: 52.4%, Nuclear and Cytoplasmic: 47.6%; NON: Nuclear: 93.3%, Nuclear and Cytoplasmic: 6.7%; MIMIC: Nuclear: 31.1%, Nuclear and Cytoplasmic: 68.9%; Figures 5A–C″), indicating that the phosphorylation of SOX11 impacts on its subcellular localization. To further specify, which serines are responsible for SOX11’s differential localization, the phosphorylatable serines were assigned to two groups regarding their position and conservation. The first group consisted of the SOX11-specific, conserved N-terminal serines surrounding the HMG box (S30, S133, S137) while the second group consisted of the C- terminal serines and putative phosphorylation sites (S244, S248, S249, S253, S258, S289, S292; Figure 3D). The respective serines of either group were mutated into a phosphomimetic or a non-phosphorylatable form to yield: (i) a mutant with the three N-terminal serines (S30, S133, S137) mutated into a phosphomimetic form (C3-Sox11p^M3W7^ = MIMIC 3 WT 7) or (ii) mutated into a non-phosphorylatable form (C3-Sox11p^N3W7^ = NON 3 WT 7), (iii) a mutant with the seven C-terminal serines (S244, S248, S249, S253, S258, S289, S292) mutated into a phosphomimetic form (C3-Sox11p^W3M7^ = WT 3 MIMIC 7) or (iv) mutated into a non-phosphorylatable form (C3-Sox11p^W3N7^ = WT 3 NON 7; Figure 3D). In contrast to the SOX11p^WT^ protein and the other mutants (Sox11p^M3W7^, Sox11p^W3M7^, Sox11p^W3N7^) which showed a nuclear and cytoplasmic localization (M3W7: Nuclear: 44.4%, Nuclear and Cytoplasmic: 55.6%; W3M7: Nuclear: 41.3%, Nuclear and Cytoplasmic: 58.7%; W3N7: Nuclear: 44.2%, Nuclear and Cytoplasmic: 55.8%; Figures E–G″), Sox11p^N3W7^ was exclusively found in the nucleus (N3W7: Nuclear: 93.2%, Nuclear and Cytoplasmic: 2.9%; Figures D–D″) suggesting that the conserved N-terminal phosphorylatable serines play a role in SOX11’s subcellular localization. The statistical analysis of the subcellular distribution of the SOX11 mutants relative to SOX11 WT is summarized in Supplementary Table S2.

To further elucidate, which of the three N-terminal serine (S30, S133, S137) phosphorylation influences SOX11’s localization, we generated: (i) mutants with S30 mutated into a phosphomimetic form (C3-Sox11p^M1W9^ = MIMIC 1 WT 9) or in a non-phosphorylatable form (C3-Sox11p^N1W9^ = NON 1 WT 9); and (ii) mutants with the two serines after the HMG box (S133, S137) mutated into a phosphomimetic form (C3-Sox11p^W1M2W7^ = WT 1 MIMIC 2 WT 7) or into a non-phosphorylatable form (C3-Sox11p^W1N2W7^ = WT 1 NON 2 WT 7). Following transfection into HEK293T cells the SOX11p^W1M2W7^ and SOX11p^W1N2W7^ mutants showed a cytoplasmic and nuclear localization that was comparable to the SOX11p^WT^ (Figures A–A″) (W1M2W7: Nuclear: 59.5%, Nuclear and Cytoplasmic: 40.5%; W1N2W7: Nuclear: 51.2%, Nuclear and Cytoplasmic: 48.8%; Figures D–E″), indicating that the two serines after the HMG box (S133, S137) did not regulate SOX11’s subcellular localization. The SOX11p^M1W9^ also localized to the cytoplasm and the nucleus while the SOX11p^N1W9^ mutant was almost completely restricted to the nucleus (M1W9: Nuclear: 26.1%, Nuclear and Cytoplasmic: 73.9%; N1W9: Nuclear: 96.2%, Nuclear and Cytoplasmic: 3.8%; Figures B–C″). The statistical analysis of the subcellular distribution of the SOX11 mutants relative to SOX11 WT is summarized in Supplementary Table S2.

**Figure 6:**
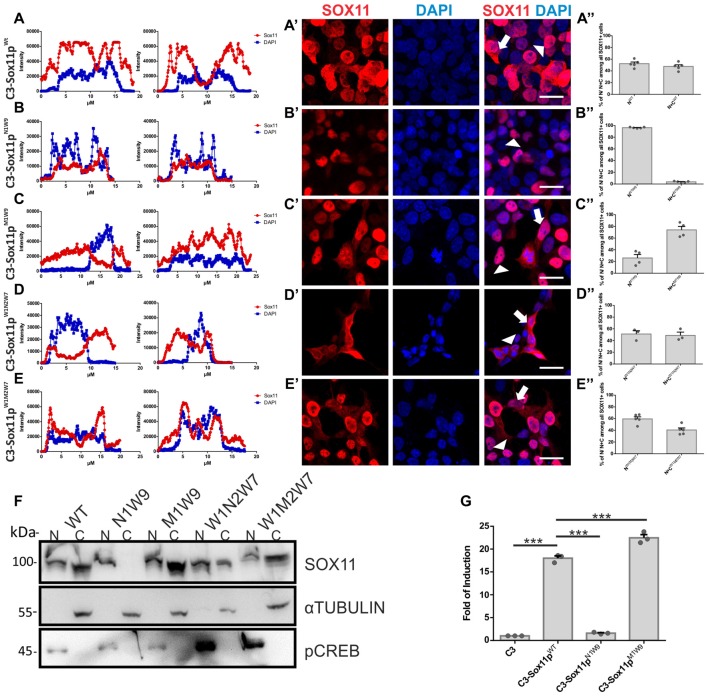
SOX11’s subcellular localization depends on the phosphorylation of S30. **(A–E)** Representative line intensity plots of HEK293T cells transfected with SOX11 WT and mutants. The blue line represents DAPI intensity, the red line represents SOX11 intensity. **(A’–E’)** Immunostaining for SOX11 (red) and DAPI (blue) of cells transfected with SOX11 WT and mutants were to determine the subcellular localization of SOX11 phospho-mutants. Note that the C3-Sox11p^N1W9^ is almost exclusively localized in the nucleus **(B’)**. Arrow: cell with nuclear and cytoplasmic localization of SOX11. Arrow head: cell with only nuclear localization of SOX11. Scale bars: 20 μm. **(A″–E″)** Percentage of cells with nuclear localization (N) or nuclear and cytoplasmic (N + C) localization of the different SOX11 mutants. **(F)** Western Blot analysis of nuclear (N) and cytoplasmic (C) extracts of Neuro2a cells overexpressing C3-Sox11p^Wt^ (WT), C3-Sox11p^N1W9^ (N1W9), C3-Sox11p^M1W9^ (M1W9), C3-Sox11p^W1N2W7^ (W1N2W7) and C3-Sox11p^W1M2W7^ (W1M2W7). First row: blotting with an anti-SOX11 antibody; Second row: Blotting with an anti-pCREB (S133) antibody validates enrichment of nuclear proteins; third row: Blotting with an anti-αTUBULIN validates enrichment of cytoplasmic proteins. Note that the N1W9 mutant is exclusively detected in the nuclear fraction. **(G)** Activity of a luciferase reporter driven by a minimal SOX11 responsive promoter in HEK293T cells overexpressing C3 (empty backbone), C3-Sox11p^Wt^, C3-Sox11p^N1W9^ and C3-Sox11p^M1W9^, *n* = 3 (***p < 0.001).

For validation of the impact of S30 phosphorylation on subcellular localization by an independent method, we overexpressed the mutants in Neuro2A cells, a mouse brain neuroblastoma cell line. Western Blot analysis of nuclear and cytoplasmic extracts from the SOX11/mutants overexpressing cells showed that while SOX11p^Wt^ and mutants (SOX11p^M1W9^, SOX11p^W1M2W7^ and SOX11p^W1N2W7^) yielded a band in both nuclear and cytoplasmic extracts, Sox11p^N1W9^ yielded a band only in the nuclear extracts (Figure 6F and Supplementary Figure S4). Collectively, these results indicate that the phosphorylation status of S30 impacts on SOX11’s subcellular localization.

Finally, to begin to understand whether the phosphorylation of S30 influences SOX11’s transcriptional activity, we performed Luciferase assays on a Sox11-responsive minimal promoter in HEK293T cells. The reporter assay showed that SOX11p^M1W9^ had significantly increased transcriptional activity compared with the WT, whereas the Sox11p^N1W9^ had almost no transcriptional activity (Figure 6G).

## Discussion

The neurodevelopmental phenotypes elicited by Sox11 loss- and gain-of-function in mice as well as the causal link between Sox11 haplo-insufficiency and the intellectual disability associated Coffin-Siris Syndrome underline the importance to precisely regulate SOX11 activity (Kavyanifar et al., 2018).

Regulation of subcellular localization is a powerful mechanism that controls the activity of a number of TFs (Cartwright and Helin, 2000). Nuclear vs. cytoplasmic localization not only determines the ability of a TF to bind to DNA, but may also be a pre-requisite for interaction with spatially-restricted co-factors that modulate a TFs function (Eijkelenboom and Burgering, 2013). Intriguingly, we found that the nuclear vs. cytoplasmic localization pattern of SOX11 changes during different stages of neural development, suggesting that SOX11 activity is regulated at least in part by regulation of its subcellular localization.

Using mass spectrometry-based identification of putative phosphorylation sites followed by mutational analysis, we identified one serine residue, i.e., S30, which strongly modulated SOX11’s subcellular localization *in vitro*. Thus, mutation of this residue to a non-phosphorylatable amino acid (S30A) resulted in an almost exclusive nuclear localization, while its phosphomimetic mutation (S30D) resulted in a nuclear and cytoplasmic localization. Contrary to the expectation that preferential nuclear localization would be associated with increased transcriptional activity, the S30A mutant failed to activate a Sox11-responsive minimal promoter whereas the S30D mutant displayed increased transcriptional activity compared to wildtype SOX11. These data indicate that nuclear localization is not sufficient to activate SOX11-dependent gene expression. The data also suggest that the transcriptional activity of SOX11 is modulated by S30 phosphorylation and that export of the transcriptionally active SOX11 to the cytoplasm may support the inactivation of SOX11-dependent gene-expression programs.

In addition to the S30 residue, mass spectrometric analysis identified nine serine residues with the potential for phosphorylation. As these residues differ in their surrounding sequences, SOX11 may be targeted by different kinases and pathways, which may result in combinatorial phosphorylation code that modulates SOX11’s activity and target specificity. It will also be interesting how phosphorylation interacts with other PTMs to regulate SOX11’s function. Recent work discovered that SUMOylation suppresses SOX11’s nuclear localization in developing retinal ganglion cells (Chang et al., 2017). Whether SUMOylation and phosphorylation co-occur and how they interact to control SOX11’s subcellular localization and function remains to be determined. Notably, we detected two lower molecular weight bands for SOX11 following phosphatase-treatment of embryonic brain extracts, which may reflect the presence of SUMOylation or other additional PTMs.

SOX11’s potential regulation by a combinatorial PTM code may help to explain the observations that SOX11 upregulation can be associated with both, good or poor prognosis in different tumor types (Weigle et al., 2005; Wang et al., 2008; Brennan et al., 2009; Kuo et al., 2015) and that distinct retinal ganglion cell populations respond to SOX11 expression with opposing phenotypes, i.e., regeneration or cell death, in the lesion context (Norsworthy et al., 2017; Welsbie et al., 2017). It will be interesting to determine whether distinct PTM codes are associated with opposing phenotypes and to elucidate the impact of distinct PTM combinations on SOX11’s activity, target specificity and interaction with co-factors. It will also be important to identify the pathways that dictate SOX11’s PTMs. Such studies may help to develop strategies to manipulate and control SOX11 function in the context of pathologies.

## Author Contributions

E-AB and DCL: conceptualization. E-AB, M-TW, MJ, BMH, BH, FZ, JH, JW, ES and CJG: investigation. E-AB, BH and DCL: formal analysis. DCL and CJG: recourses and funding, acquisition and supervision. E-AB, CJG and DCL: writing-original draft, writing-review and editing.

## Conflict of Interest Statement

The authors declare that the research was conducted in the absence of any commercial or financial relationships that could be construed as a potential conflict of interest.

## Supplementary Material

FIGURE S1 Validation of the anti-SOX11 antibody specificity in immunofluorescent stainings **(A–D)**. Coronal sections of E18.5 wildtype (wt) and knockout (ko) mouse brain were stained for SOX11 (red), TUJ1 (green) and DAPI (blue). The E18.5wt shows strong SOX11 immunoreactivity with a clear nuclear and cytoplasmic signal. Note the absence of SOX11 immunoreactivity in the E18.5ko, Scale bars: 50 μm. **(E)** SOX11’s subcellular localization in embryonic neurogenesis. Coronal sections of brains from E11.5 to P0 were stained for DAPI (blue), to mark the nuclei. White boxes indicate the cortex areas in which the images from Figure 1 were taken. Scale bar: 100 μm. Line intensity plot of example cells with an exclusive nuclear **(F–H)** and a nuclear and cytoplasmic SOX11 distribution **(I–K)**. HEK293T3 cells overexpressing the WT Sox11:C3-Sox11p^Wt^, were stained for SOX11 (red) to analyze its subcellular localization. The upper transfected cell has a nuclear localization of SOX11. The intensity plot of the SOX11 signal was generated by drawing a line in ImageJ and by applying the Plot Profile function. The same line was used to measure DAPI’s intensity **(G)**. Overlay of the intensity plots **(H)** showed that SOX11 intensity decreases completely when DAPI’s intensity decreases indicating SOX11’s nuclear localization. The bottom cell was analyzed accordingly. Note that in this sample cell, SOX11’s intensity remains high even when DAPI’s intensity reaches almost zero indicating that SOX11 localizes to the nucleus and cytoplasm.

FIGURE S2 **(A–C)** Subcellular Localization of SOX11 is region dependent. In contrast to the E15.5 cortex, in which SOX11 is almost exclusively nuclear (Figures 1A–A″ and Supplementary Figure 1E), SOX11 is found in the nucleus and the cytoplasm of cells in subcortical regions (Scale bars: 100 μm). The white box on **(A)** depicts the area in which the higher magnification images of **(B,C)** were taken. Arrows: cells with nuclear and cytoplasmic localization of SOX11 analyzed by line intensity plots **(B′,C′)**. **(D–F)** HEK293T were transfected with the CAG-Sox11-IRES-GFP plasmid to overexpress non-tagged wildtype SOX11. Staining with anti-SOX11 antibody (red) and DAPI (blue) as a nuclear marker shows that the non-tagged SOX11 can localize to both nucleus and cytoplasm. **(E)** Percentage of cells with nuclear localization (N) or nuclear and cytoplasmic (N + C) localization of wildtype SOX11. **(F)** The line intensity plot depicts example cells with an exclusive nuclear and a nuclear and cytoplasmic SOX11 distribution. Scale bar: 20 μm.

FIGURE S3 **(A)** Experimental work flow for the generation of Phospho Stop and lambda phosphatase treated nuclear and cytoplasmic extracts from embryonic mouse brains. **(B–G)** Full blots from Figure 3A. The antibody used for blotting is listed below the blot.

FIGURE S4 Full blots from Figure 6F. **(A)** Blotting against SOX11, pRNApolymerase II as a nuclear marker, and against αTubulin as a cytoplasmic marker. **(B)** Blotting against pCREB as a nuclear marker.

TABLE S1 The table summarizes the mass spectrometric analysis. The peptide report (filtered for Sox11 phospho peptides) of Proteome Discoverer is provided. In addition to the standard information (including the confidence level and identification scores), it also reports site-probability sores for the observed phosphorylation determined by the Proteome Discoverer Module PhosphoRS. In addition, the cell type (HEK293T or Neuro2a) and fraction (nuclear/NE or cytosolic/CE) are provided for each peptide. Two individual sets of experimental data from two different mass spectrometers (fragmentation and detection methods) are shown: FVZ1262 (ITMS/Orbitrap Fusion, CID fragmentation/linear ion trap detection) and FVZ2070 (FTMS/Q-Exactive Plus, HCD fragmentation/Orbitrap detection). Furthermore, Representative spectra for each phospho peptide isoform are provided.

TABLE S2 Statistical analysis of the subcellular distribution of each SOX11 mutant relative to SOX11 WT. Data from Figures 5, 6 were analyzed for their significant differences with multiple comparison One-Way ANOVA, carried out with GraphPad Prism. The statistical comparisons of the samples are represented by (*) for significant differences and with (ns) for not significant differences as also shown by the given *p*-values (**p* < 0.05, ***p* < 0.01, ****p* < 0.001).
