# Supplementary figures and images for: Phosphorylation Modulates the Subcellular Localization of SOX11
Source: Front Mol Neurosci. 2018 Jun 19;11:211. doi: 10.3389/fnmol.2018.00211 (PMC6020773; doi:10.3389/fnmol.2018.00211)

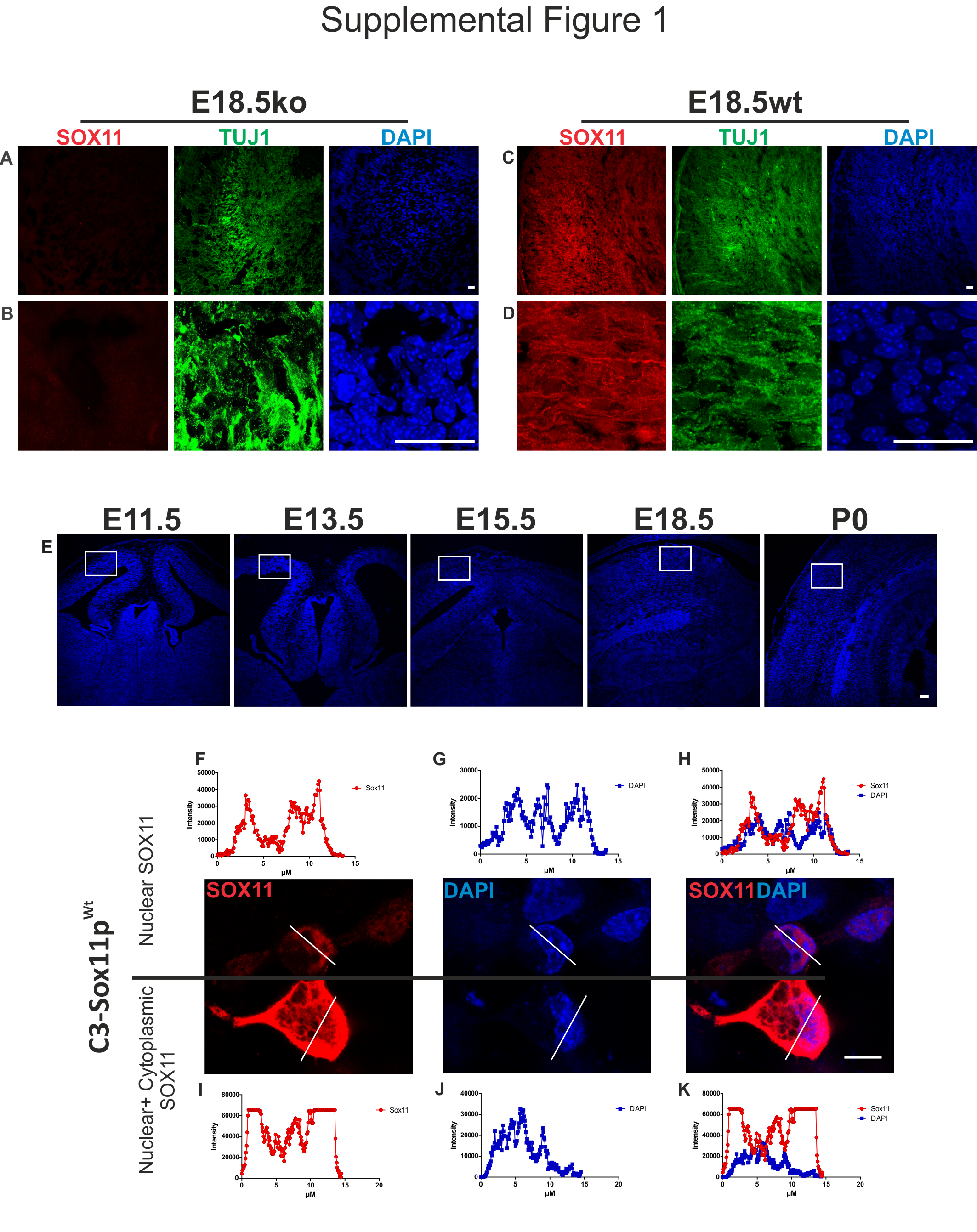

Supplement: FIGURE S1 — Validation of the anti-SOX11 antibody specificity in immunofluorescent stainings (A–D). Coronal sections of E18.5 wildtype (wt) and knockout (ko) mouse brain were stained for SOX11 (red), TUJ1 (green) and DAPI (blue). The E18.5wt shows strong SOX11 immunoreactivity with a clear nuclear and cytoplasmic signal. Note the absence of SOX11 immunoreactivity in the E18.5ko, Scale bars: 50 μm. (E) SOX11’s subcellular localization in embryonic neurogenesis. Coronal sections of brains from E11.5 to P0 were stained for DAPI (blue), to mark the nuclei. White boxes indicate the cortex areas in which the images from Figure 1 were taken. Scale bar: 100 μm. Line intensity plot of example cells with an exclusive nuclear (F–H) and a nuclear and cytoplasmic SOX11 distribution (I–K). HEK293T3 cells overexpressing the WT Sox11:C3-Sox11pWt, were stained for SOX11 (red) to analyze its subcellular localization. The upper transfected cell has a nuclear localization of SOX11. The intensity plot of the SOX11 signal was generated by drawing a line in ImageJ and by applying the Plot Profile function. The same line was used to measure DAPI’s intensity (G). Overlay of the intensity plots (H) showed that SOX11 intensity decreases completely when DAPI’s intensity decreases indicating SOX11’s nuclear localization. The bottom cell was analyzed accordingly. Note that in this sample cell, SOX11’s intensity remains high even when DAPI’s intensity reaches almost zero indicating that SOX11 localizes to the nucleus and cytoplasm. [file Image_1.TIF]

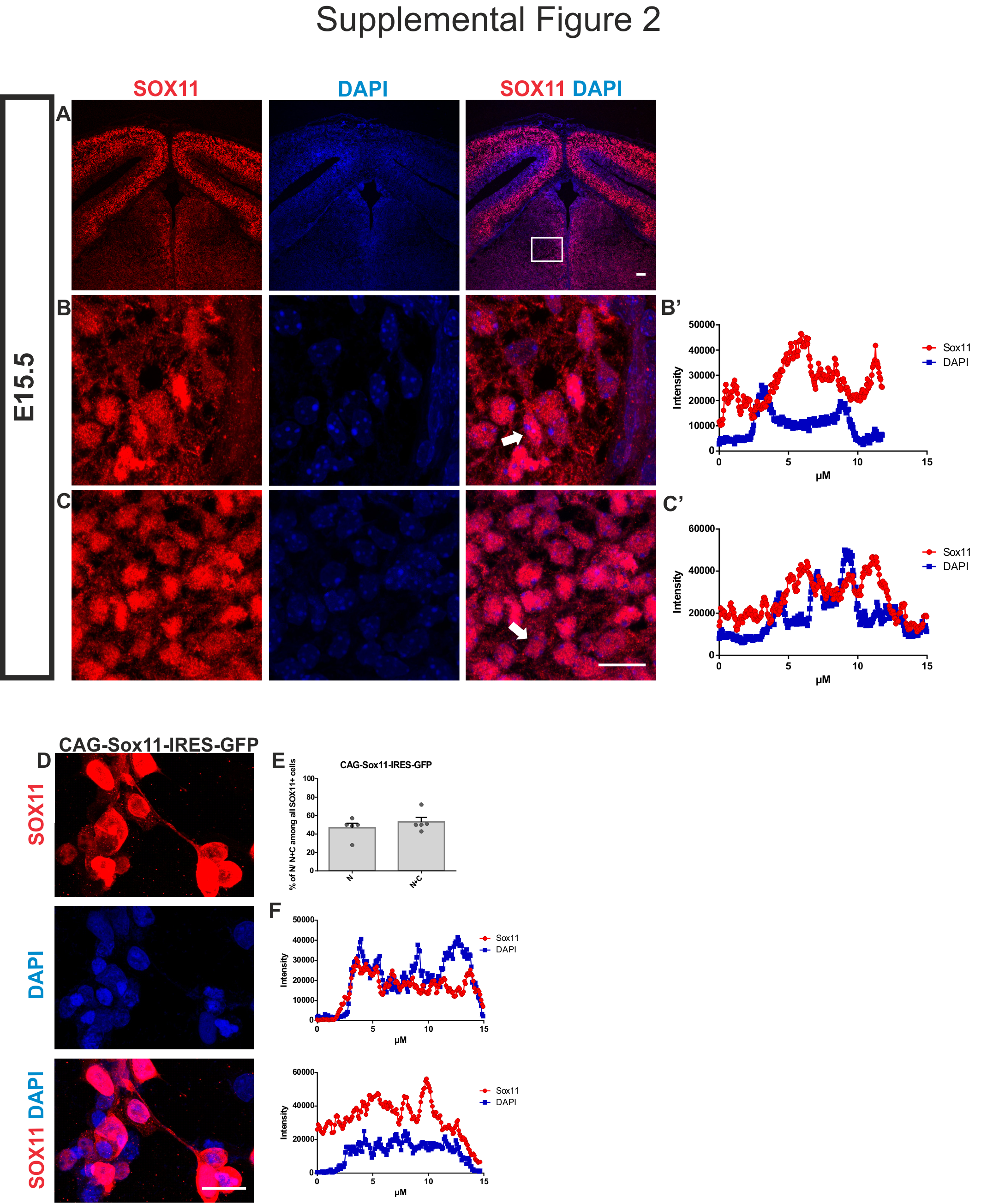

Supplement: FIGURE S2 — (A–C) Subcellular Localization of SOX11 is region dependent. In contrast to the E15.5 cortex, in which SOX11 is almost exclusively nuclear (Figures 1A–A″ and Supplementary Figure 1E), SOX11 is found in the nucleus and the cytoplasm of cells in subcortical regions (Scale bars: 100 μm). The white box on (A) depicts the area in which the higher magnification images of (B,C) were taken. Arrows: cells with nuclear and cytoplasmic localization of SOX11 analyzed by line intensity plots (B′,C′). (D–F) HEK293T were transfected with the CAG-Sox11-IRES-GFP plasmid to overexpress non-tagged wildtype SOX11. Staining with anti-SOX11 antibody (red) and DAPI (blue) as a nuclear marker shows that the non-tagged SOX11 can localize to both nucleus and cytoplasm. (E) Percentage of cells with nuclear localization (N) or nuclear and cytoplasmic (N + C) localization of wildtype SOX11. (F) The line intensity plot depicts example cells with an exclusive nuclear and a nuclear and cytoplasmic SOX11 distribution. Scale bar: 20 μm. [file Image_2.TIF]

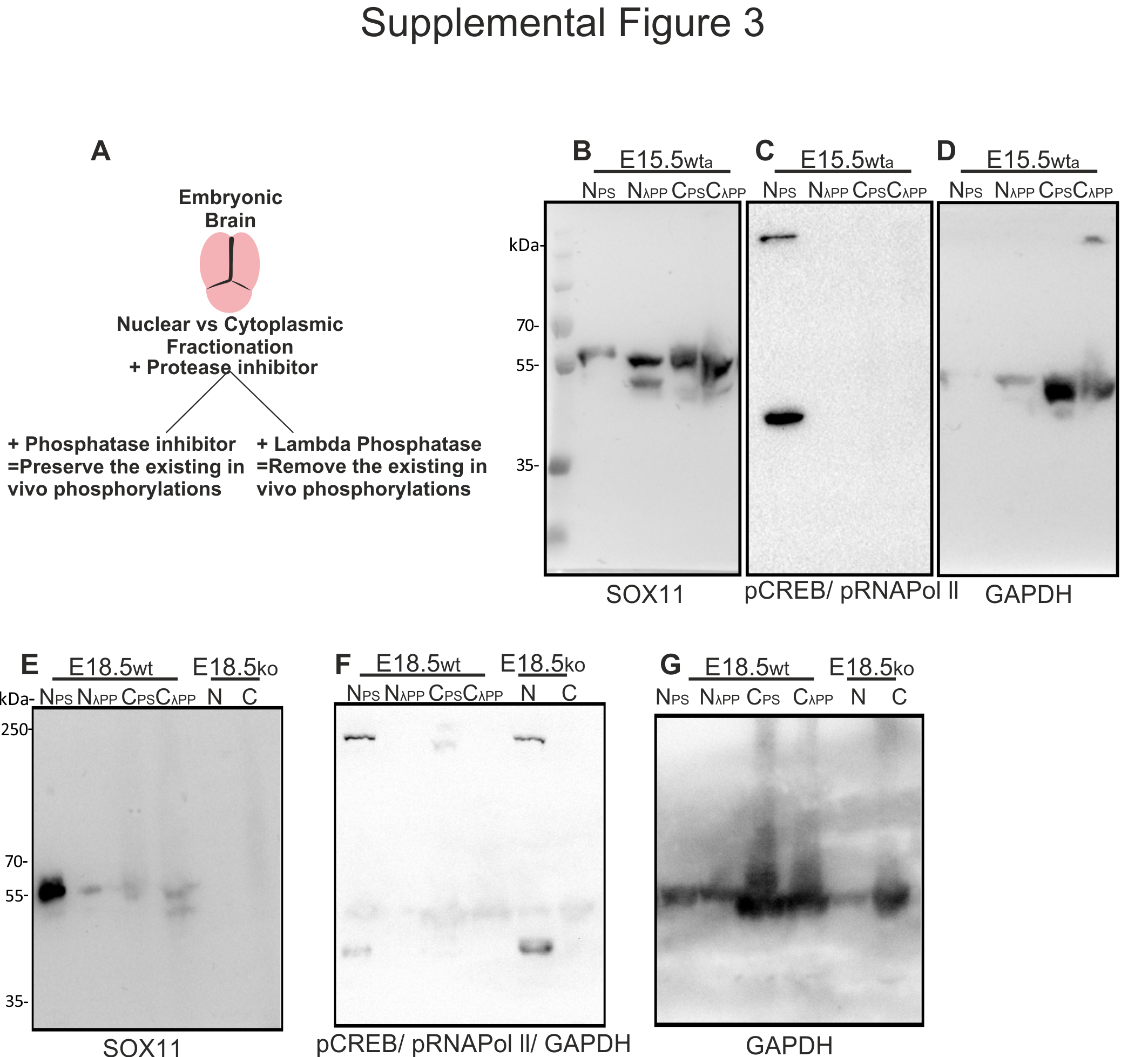

Supplement: FIGURE S3 — (A) Experimental work flow for the generation of Phospho Stop and lambda phosphatase treated nuclear and cytoplasmic extracts from embryonic mouse brains. (B–G) Full blots from Figure 3A. The antibody used for blotting is listed below the blot. [file Image_3.TIF]

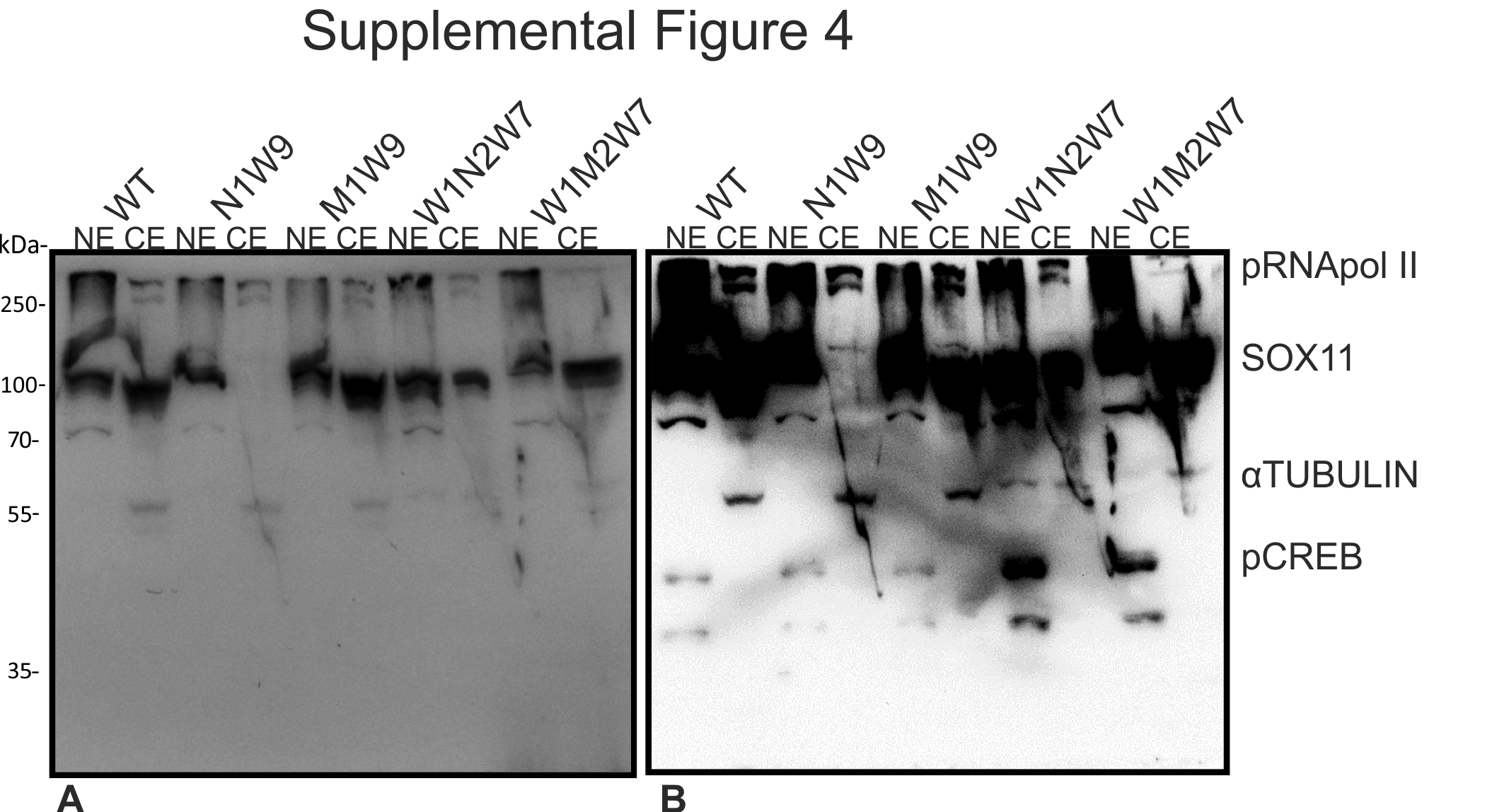

Supplement: FIGURE S4 — Full blots from Figure 6F. (A) Blotting against SOX11, pRNApolymerase II as a nuclear marker, and against αTubulin as a cytoplasmic marker. (B) Blotting against pCREB as a nuclear marker. [file Image_4.TIF]
